# Successful Transcatheter Aortic Valve Implantation for Severe Aortic Stenosis in Congenitally Corrected Transposition of the Great Arteries

**DOI:** 10.1016/j.cjco.2026.06.012

**Published:** 2026-06-29

**Authors:** Miguel Sobral Domingues, Rita Almeida Carvalho, João Brito, Rui Anjos, Henrique Mesquita Gabriel, Rui Campante Teles, Sérgio Madeira

**Affiliations:** a Interventional Cardiology Department, Hospital de Santa Cruz -Unidade Local de Saúde de Lisboa Ocidental (ULSLO) - Centro Clínico Académico de Lisboa (CCAL), Carnaxide, Portugal; b Pediatric Cardiology Department, Hospital de Santa Cruz - ULSLO - CCAL, Carnaxide, Portugal; c Adult Congenital Heart Disease Department, Hospital de Santa Cruz - ULSO - CCAL, Carnaxide, Portugal

**Keywords:** adult congenital heart disease, congenital heart disease, transcatheter aortic valve, transcatheter aortic valve implantation

**Congenitally corrected transposition of the great arteries is a rare congenital heart disease characterized by atrioventricular and ventriculoarterial discordance. Degenerative severe aortic stenosis is exceptionally uncommon in this population because many patients develop systemic right ventricular dysfunction before reaching advanced age. We report the case of a 77-year-old woman with congenitally corrected transposition and previous tricuspid valve replacement who developed *de novo* high-gradient severe aortic stenosis and was successfully treated with transfemoral transcatheter aortic valve implantation after extensive multimodality imaging and multidisciplinary heart team evaluation.**

Congenitally corrected transposition of the great arteries (ccTGA) accounts for less than 1% of congenital heart diseases.^1^ The condition is characterised by atrioventricular and ventriculoarterial discordance, resulting in a physiologically corrected circulation in which the morphologic right ventricle supports the systemic circulation. Associated cardiac abnormalities are common and include ventricular septal defects, pulmonary outflow tract obstruction, and abnormalities of the systemic atrioventricular (tricuspid) valve. The natural history of ccTGA is frequently marked by progressive systemic right ventricular dysfunction and heart failure, limiting long-term survival. Although aortic valve disease may occur in this setting, it most commonly presents as regurgitation, whereas severe aortic stenosis is exceedingly rare and usually is associated with bicuspid valve anatomy.^1^^,^^2^ Consequently, age-related degenerative calcific aortic stenosis is exceptionally uncommon in this population.

## Patient Presentation

A 77-year-old woman with ccTGA, a restrictive ventricular septal defect, and a bioprosthetic tricuspid valve, implanted at age 65 years for severe regurgitation, presented with several months of worsening fatigue, dyspnoea on minimal exertion, and orthopnea. Additional history included permanent atrial fibrillation, stage IIIb chronic kidney disease, and stage B chronic lymphocytic leukemia under observation.

## Initial Work-Up

Transthoracic echocardiography revealed severe calcific tricuspid aortic stenosis with a mean transvalvular gradient of 60 mm Hg and a left ventricular outflow tract to aortic velocity-time integral (LVOT-to-aortic velocity-time integral) ratio of 0.14, consistent with high-gradient severe aortic stenosis (AS). Mild aortic regurgitation and moderate systemic right ventricular dysfunction were observed, but function of the left-sided atrioventricular bioprosthesis remained preserved.

Electrocardiography demonstrated atrial fibrillation at 65 beats per minute with right bundle branch block. Laboratory tests showed mild anemia (hemoglobin 11.6 g/dL), an elevated N-terminal pro-brain natriuretic peptide (NT-proBNP) level (34,461 pg/mL), and stable chronic kidney disease with a baseline glomerular filtration rate of 43 mL/min per 1.73 m^2^.

Cardiac computed tomography (CT) angiography confirmed severe calcific aortic stenosis (Agatston calcium score 2351 AU) and provided detailed characterization of the complex congenital anatomy. The systemic ventricular outflow tract demonstrated a thick-walled, predominantly muscular subvalvular region without aneurysmal dilation or funnel-shaped morphology, although with marked systolic-to-diastolic dimensional variability.

**Figure 1:**
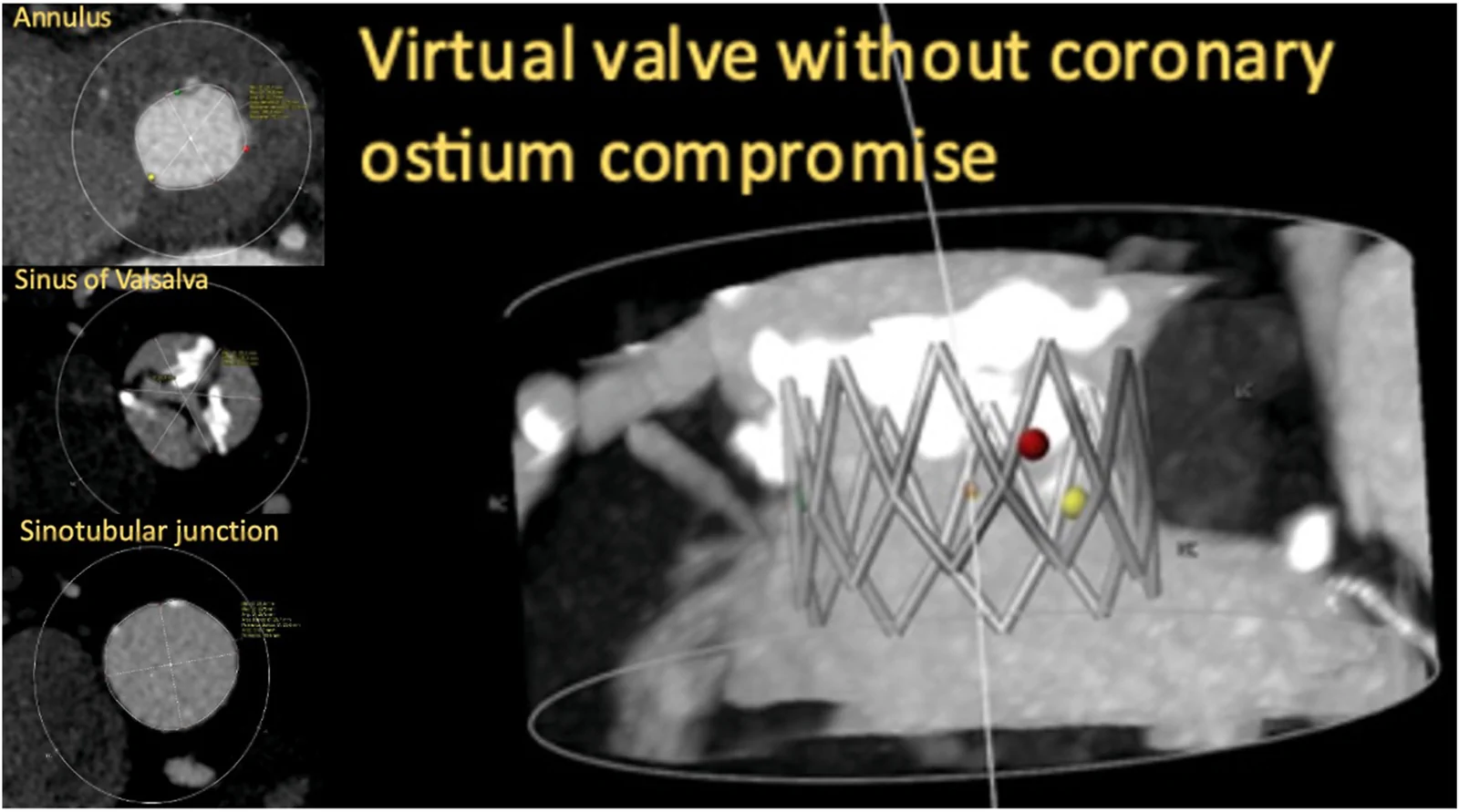
Computed tomography analysis.

Annular measurements obtained during systole were as follows (Fig. 1):

annulus diameter: 21 × 25 mm (mean diameter 23 mm);
perimeter-derived diameter: 23 mm;
perimeter: 72 mm;
annular area: 397 mm^2^;
sinuses of Valsalva dimensions: 25 × 26 × 27 mm; and
sinotubular junction: 25 × 28 mm (mean 26.5 mm).

Coronary heights were low, measuring 7 mm for the right coronary artery and 8 mm for the left main coronary artery.

Coronary anatomy was abnormal but consistent with typical ccTGA coronary distribution. The dominant right coronary artery originated from the left-facing sinus and coursed within the left atrioventricular groove supplying the systemic right ventricle. The left main coronary artery arose from the right-facing sinus and bifurcated into the left anterior descending artery, which coursed in the anterior interventricular groove, and a circumflex artery running within the right atrioventricular groove.

Despite the absence of a well-defined fibrous annulus and the presence of a thick-walled, muscular, and dynamic systemic ventricular outflow tract, the CT analysis suggested the feasibility and safety of transcatheter valve implantation.

## Surgical Risk Assessment

Given the patient’s age, previous cardiac surgery, chronic kidney disease, moderate systemic right ventricular dysfunction, and the anticipated technical complexity associated with redo surgery in ccTGA, surgical risk was considered very high (**Euro**pean **S**ystem for **C**ardiac **O**perative **R**isk **E**valuation [EuroSCORE] II 14.2%; Society of Thoracic Surgeons predicted operative mortality 9.2%, and estimated morbidity and mortality 25.5%).

Following multidisciplinary heart team discussion involving structural interventional cardiologists, cardiac imaging specialists, and a congenital cardiac surgeon experienced in adult congenital heart disease, a transfemoral transcatheter approach was favoured.

## Device Selection and Management

**Figure 2:**
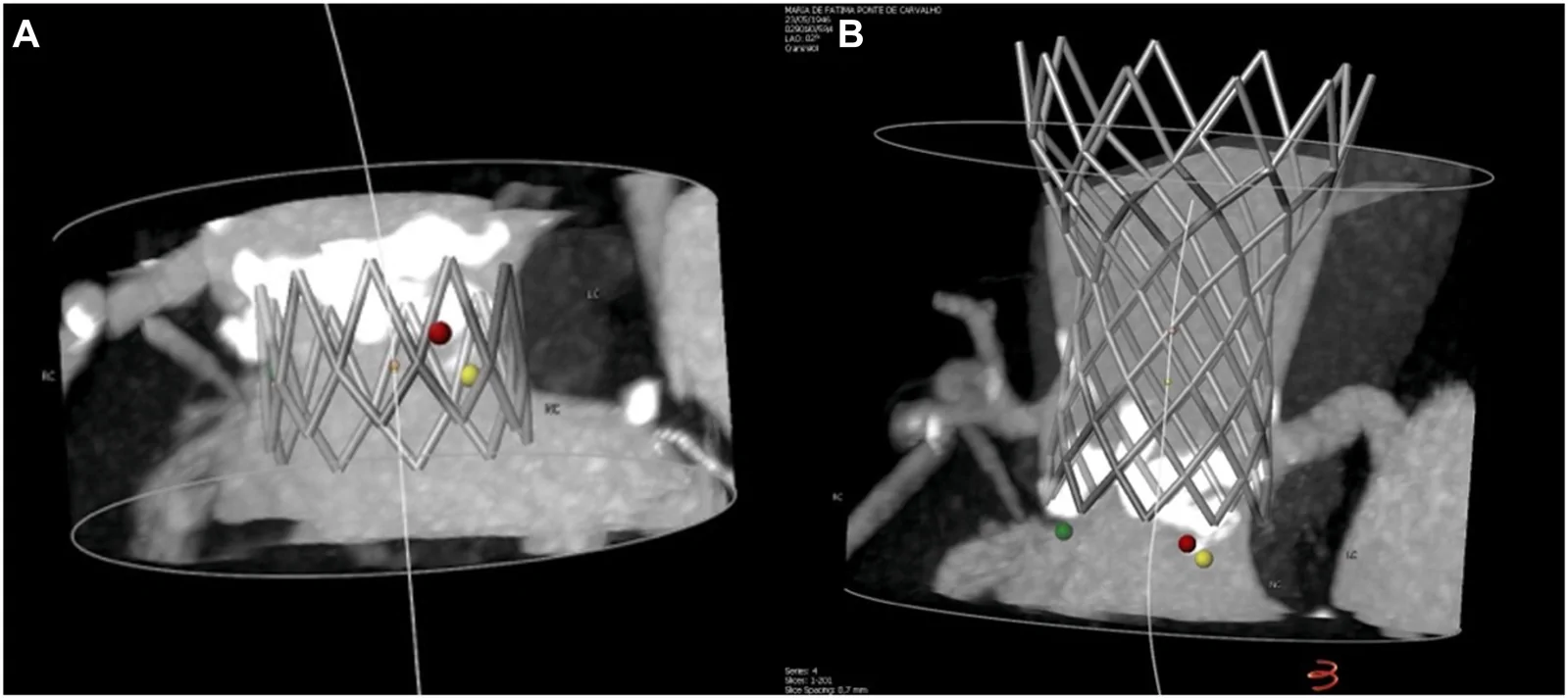
Computed tomography–based virtual simulation of: (**A**) balloon expandable valve; and (**B**) self-expandable valve.

A balloon-expandable short-frame valve was selected instead of a self-expandable valve (Fig. 2.), to optimise deployment precision in the setting of a poorly defined muscular annulus with substantial dynamic variability, low coronary heights (right coronary 7 mm, left coronary 8 mm), and narrow sinuses of Valsalva (25 × 26 × 27 mm) and sinotubular junction (25 × 28 mm). A 23-mm Edwards Sapien Ultra (Edwards Lifesciences, Irvine, CA) matched the annular dimensions (annular area 397 mm^2^, perimeter-derived diameter 23 mm) and allowed implantation at 2 mm depth while preserving access to the coronary ostia (Fig. 1.). The interplay between device dimensions (valve height 18 mm, inner skirt height 9.3 mm, open-cell frame ∼9 mm), depth of implantation, leaflet dimensions, and surrounding anatomy—yielding an acceptable virtual valve-to-coronary (VTC) distance on CT—precluded the need for coronary protection techniques. Pre-dilatation was performed with a 20-mm Nucleus balloon (NuMED, Inc., Hopkinton, NY), followed by valve implantation at nominal balloon volume (Video 1, view video online) and post-dilatation with the valve balloon plus 1 mL to correct a mild residual paravalvular leak, yielding excellent prosthesis expansion, a single-digit gradient, and no regurgitation. This strategy was designed to minimize the risks of valve embolization, deep implantation, conduction disturbance, and coronary obstruction.

## Follow-Up

No vascular access or rhythm complications occurred. Post-procedural transthoracic echocardiography confirmed normal transvalvular gradients without residual paravalvular leak, and the patient was discharged uneventfully on the sixth day post–transcatheter aortic valve implantation (TAVI). During follow-up, she showed marked improvement in symptoms, from New York Heart Association class III to I, and remained clinically stable at 1-year follow-up.

## Discussion

Reports of TAVI in patients with congenitally corrected transposition of the great arteries and aortic valve disease are exceedingly rare. To date, only isolated cases have been described, including one patient with low-flow, low-gradient severe aortic stenosis, and 2 additional cases of degenerative aortic stenosis reported within broader case series without detailed procedural characterization.^3^, ^4^, ^5^ To our knowledge, this paper represents the first detailed report of successful transcatheter treatment of degenerative high-gradient severe aortic stenosis in an elderly patient with ccTGA.

The rarity of this clinical scenario likely reflects the natural history of ccTGA. Progressive dysfunction of the systemic right ventricle and systemic atrioventricular valve frequently limits long-term survival, such that many patients do not survive to the age at which degenerative calcific aortic stenosis typically develops.^1^ This case also highlights the increasing longevity of patients with adult congenital heart disease and the growing need to address acquired degenerative valvular disease in this expanding population.

This case emphasizes several important anatomic and procedural considerations relevant to transcatheter aortic valve implantation in complex congenital heart disease. Pre-procedural cardiac CT demonstrated a thick-walled, predominantly muscular systemic ventricular outflow tract with marked dimensional variation between systole and diastole and no well-defined fibrous annulus. Such anatomy complicates annular sizing, definition of the anchoring target, and the depth of implantation. In addition, low coronary heights and narrow sinuses of Valsalva raised the theoretical risk of coronary obstruction after valve deployment. The coronary distribution, although atypical compared with that of a normal heart, was consistent with the pattern expected in congenitally corrected transposition of the great arteries. Careful evaluation of coronary orientation and its relationship to the annulus and valve leaflets was essential during procedural planning.

In this context, a short-frame balloon-expandable valve was selected to optimize deployment precision and minimize the risks of valve embolization, coronary obstruction, deep implantation, and atrioventricular conduction disturbance. Despite the low coronary heights, coronary protection was not deemed necessary, as pre-procedural imaging demonstrated an acceptable relationship between leaflet dimensions and the coronary ostia, as well as an adequate valve-to-coronary (VTC) distance.

An important point to note is that conventional surgical risk scores may incompletely capture the procedural complexity associated with adult congenital heart disease. However, in the present case, both the EuroSCORE II and the Society of Thoracic Surgeons risk estimates were markedly elevated, further supporting a less invasive strategy. Multidisciplinary heart team assessment involving both congenital and structural heart disease specialists was fundamental to procedural planning and successful treatment.

This case suggests that TAVI may represent a feasible therapeutic option in carefully selected patients with ventriculoarterial discordance and aortic stenosis when meticulous multimodality imaging, individualized procedural planning, and highly experienced operators are available. Further experience is required to better define patient selection, procedural strategies, and long-term outcomes in this rare clinical setting.

**Novel Teaching Points**

• Degenerative severe aortic stenosis is exceptionally rare in congenitally corrected transposition of the great arteries because many patients develop systemic right ventricular failure before reaching advanced age.
• Pre-procedural multimodality imaging is critical when planning TAVI in complex congenital heart disease anatomy.
• Careful heart team assessment involving congenital and structural heart specialists is essential when considering transcatheter therapy in adult congenital heart disease patients.
• Balloon-expandable transcatheter valves may provide procedural advantages in selected patients with complex congenital ventricular outflow tract anatomy.

## Ethics Statement

The research repoorted im this paper adhered to the CARE guidelines.

## Patient Consent

The authors confirm that patient consent was not applicable to this retrospective case report using fully de-identified clinical and imaging data, in accordance with institutional policy.

## Funding Sources

The authors have no funding sources to declare.

## Disclosures

The authors have no conflicts of interest to disclose.

## Supplementary Material

Video 1 balloon expandable valve implantation
